# Effect of Age on Clinical Outcomes Following On-/Off-Pump Coronary Artery Bypass: Meta-Analysis and Meta-Regression

**DOI:** 10.21470/1678-9741-2018-0388

**Published:** 2020

**Authors:** Hayley Mauldon, Gudrun Dieberg, Neil Smart, Nicola King

**Affiliations:** 1 University of Plymouth, Ringgold Standard Institution, Plymouth, United Kingdom of Great Britain and Northern Ireland.; 2 University of New England, Ringgold Standard Institution, Armidale, Australia.

**Keywords:** Cardiopulmonary Bypass, Coronary Artery Bypass, Off-Pump, Myocardial Infarction, Stroke, Comorbidity, Incidence

## Abstract

**Objective:**

There is currently much debate about which patients would benefit more after on- or off-pump coronary artery bypass grafting (CABG). The aim of this meta-analysis and meta-regression is to investigate the effect of age on short-term clinical outcomes after these approaches.

**Methods:**

To identify potential studies, systematic searches were carried out in the Excerpta Medica dataBASE (EMBASE), PubMed, Web of Science, and the Cochrane Central Register of Controlled Trials (CENTRAL). The search strategy included the key concepts of “cardiopulmonary bypass” AND “coronary artery bypass grafting” AND “off pump” OR “on pump”. This was followed by a meta-analysis and meta-regression investigating the effect of age on the incidences of stroke, myocardial infarction (MI), and mortality.

**Results:**

Thirty-seven studies including 15,324 participants were analysed. Overall, there was a significant odds reduction for patients receiving off-pump CABG suffering a stroke (odds ratio [OR] 0.770, 95% confidence intervals [CI] 0.594, 0.998, *P*=0.048); however, when patients were subdivided according to different age bands, this difference disappeared. There were also no significant differences in the odds of mortality (OR 0.876, 95% CI 0.703, 1.093, *P*=0.241) or MI (OR 0.937, 95% CI 0.795, 1.105, *P*=0.439). Meta-regression analysis revealed no significant relationship between age and stroke (*P*=0.652), age and mortality (*P*=548), and age and MI (*P*=0.464).

**Conclusion:**

Patients undergoing CABG are becoming older and may suffer from multiple comorbidities increasing their risk profile. However, with respect to short-term clinical outcomes, the patient’s age does not help in determining whether off- or on-pump is superior.

**Table:** 

Abbreviations, acronyms & symbols				
**ARDS**	**= Acute respiratory distress syndrome**		**MDA**	**= Malondialdehyde**
**CABG**	**= Coronary artery bypass grafting**		**MI**	**= Myocardial infarction**
**CENTRAL**	**= Cochrane Central Register of Controlled Trials**		**MRI**	**= Magnetic resonance imaging**
**CI**	**= Confidence intervals**		**N/A**	**= Not available.**
**CK-MB**	**= Creatine kinase-muscle/brain**		**NR**	**= Not reported**
**CMA**	**= Comprehensive Meta-Analysis**		**NT-proBNP**	**= N-terminal pro b-type natriuretic peptide**
**CPB**	**= Cardiopulmonary bypass**		**OR**	**= Odds ratio**
**cTnI**	**= Cardiac troponin I**		**PRISMA**	**= Preferred Reporting Items for Systematic Reviews and Meta-analyses**
**EMBASE**	**= Excerpta Medica dataBASE**		**RCT**	**= Randomised controlled trials**
**GOPCABE**	**= German Off-Pump Coronary Artery Bypass Grafting in Elderly Patients**		**UK**	**= United Kingdom**
**hs-CRP**	**= High-sensitivity creatine phosphate**		**USA**	**= United States of America**
**ICU**	**= Intensive care unit**			

## INTRODUCTION

Coronary artery bypass grafting (CABG) is the gold standard treatment for patients with complex coronary artery disease. Originally in the 1950s, this surgery was carried out on-pump with cardiopulmonary bypass (CPB); however, this approach can be associated with aortic damage, myocardial ischaemic injury, renal damage, coagulation disorders, and systemic pro-inflammatory responses^[1]^. In addition, the use of side biting clamps can cause the embolization of atherosclerotic material leading to neurological events. To overcome these problems, off-pump CABG was introduced in the early 1960s, which reduces the amount of aortic manipulation. This approach has problems, the surgery is more technically challenging and there can be limitations associated with graft patency, completeness of revascularisation, and repeat revascularisation requirement^[1]^. The controversy as to which approach is superior has not been resolved by recent meta-analyses^[2-4]^.

Recently, a meta-analysis was published investigating the long-term outcomes of on- *vs*. off-pump CABG^[5]^. The accompanying editorial comment suggested that the discussion should be refocused from comparing each approach overall to investigating precisely which groups of patients would benefit more from which technique^[6]^. In this respect, one group of interest is elderly people. The age of patients undergoing CABG is continually rising as a result of an increasingly aged population and improved survival rates following diagnoses^[7]^. For example, Ozen et al.^[8]^ found out that octogenarians continue to have a higher morbidity and mortality rate following CABG than younger populations. Thus, highlighting the need for investigation into the most beneficial techniques within older generations.

Yuksel et al.^[9]^ studied patients with age of >70 years and concluded that there was no significant benefit of either technique in terms of postoperative complications and mortality. However, they did find out that off-pump CABG required significantly less transfused blood products. One of the largest studies to date that included 2,539 participants with 75 years or older was the German Off-Pump Coronary Artery Bypass Grafting in Elderly Patients (GOPCABE) trial^[10]^. Again, this study found no difference between off-pump and on-pump CABG in elderly patients in terms of mortality, stroke, or MI as well as repeat revascularisation or new renal-replacement therapy after surgery. There have also been three meta-analyses investigating the effects of on- *vs*. off-pump CABG in patients with age of >70 or >80 years. The results are contradictory, *e.g*., Altarabsheh et al.^[11]^ found higher rates of stroke following on-pump surgery, whilst Panesar et al.^[12]^ and Zhu et al.^[13]^ found comparable rates. Although elderly people represent an important subset of patients, there is a much broader age range of patients undergoing on- or off-pump CABG. Therefore, the aim of this novel meta-analysis is to investigate the effect of on- *vs*. off-pump CABG on short-term clinical outcomes across the full age range of patients using both meta-analysis and meta-regression.

## METHODS

This analysis was planned in accordance with the current guidelines for performing comprehensive systematic reviews and meta-analysis with meta-regression, including the Preferred Reporting Items for Systematic Reviews and Meta-analyses (PRISMA) guidelines^[14]^.

### Search Strategy

To identify potential studies, systematic searches were carried out using the following databases: Excerpta Medica dataBASE (EMBASE), PubMed, Web of Science, and Cochrane Central Register of Controlled Trials (CENTRAL). The search was supplemented by scanning the reference lists of eligible studies. The search strategy included the key concepts of “cardiopulmonary bypass” AND “coronary artery bypass grafting” AND “off pump” OR “on pump” (Supplementary Figure 1). All identified papers were assessed independently by two reviewers (authors HM and NK). A third reviewer (author NS) was consulted to resolve disputes. Searches of published papers were conducted up until July 2018.

### Types of Studies Included

This meta-analysis and meta-regression only included randomised controlled trials (RCT) comparing patients undergoing on- *vs.* off-pump CABG. There were no language restrictions. Animal studies, review papers, and non-randomised controlled trials were excluded. Studies that did not have any of the desired outcome measures or participants who were treated by other modalities, such as percutaneous coronary intervention, were excluded. Incomplete data or data from an already included study were excluded. Studies that included interventions other than off-pump *vs.* on-pump CABG were excluded. Studies where the mean ages of patients in each group were in different age bands were excluded. Studies where there were no mortality, strokes, or myocardial infarctions (MI) rates, leading to an incalculable odds ratio (OR), were excluded.

### Participants/Population

This meta-analysis analysed RCTs of both male and female adult (≥18 years old) patients with coronary artery disease who were undergoing either off- or on-pump CABG. Other treatment modalities and interventions for coronary artery disease, such as percutaneous coronary intervention, were excluded.

### Intervention(S), Exposure(S)

This meta-analysis considered all RCTs where patients with stable angina or acute coronary syndrome were treated with either on-pump or off-pump CABG. More specifically, all RCTs where the intervention of carrying out CABG without the use of CPB were performed.

### Comparator(S)/Control

The studies in this analysis compared off-pump CABG with a usual care control group receiving on-pump CABG.

### Search Results

**Fig. 1:**
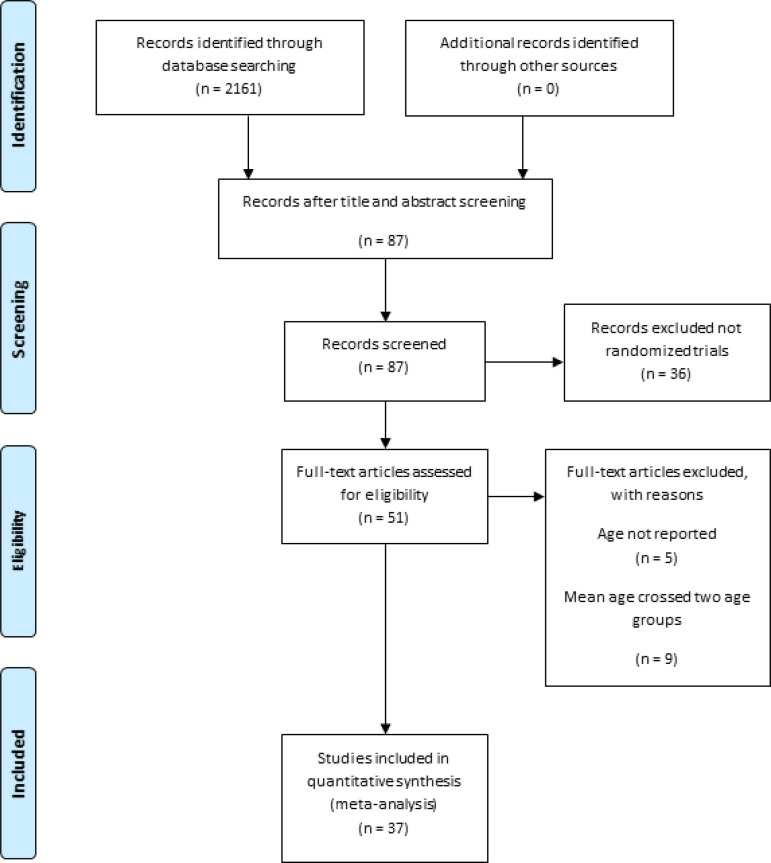
Consort figure. A flow diagram showing how the initial search results were refined until a group of studies that met all the inclusion criteria were found.

Our initial search found 2,161 articles. Of these, 2,074 studies were excluded based on title and abstract and 36 studies were excluded as they were not RCTs. Of the RCTs, we excluded 14 studies, because either they had not reported the age of the patients or the mean age of the patients crossed two age bands (Figure 1). Thirty-seven studies were included in our analysis [S1-S37].

### Outcome(S)

The primary outcomes analysed were short-term (<30 days) incidences of stroke, mortality, and MI.

### Risk of Bias (Quality) Assessment

Risk of bias was assessed using a modification of the Jadad scale^[15]^.

### Strategy for Data Synthesis

Data was collected by two authors and independently verified by a third author using pre-established tables. Patients were divided into 5-year age groups beginning at 51-55 and ending at 76-80 and investigated in their individual groups using subgroup analysis. All meta-analysis data was dichotomous and calculated as OR. An OR is a measure of association between an exposure and an outcome. The OR represents the odds that an outcome will occur given a particular exposure, compared to the odds of the outcome occurring in the absence of that exposure. Heterogeneity was quantified using the Cochrane Q test^[16]^, where I^2^=0% represents no heterogeneity and I^2^=100% represents considerable heterogeneity. A random-effects inverse variance model was used throughout. All meta-regression data was plotted as the log OR *vs*. the mean age of the patients in the off-pump group. In these graphs, a negative log OR favours off-pump and a positive log OR favours on-pump. We used a 5% level of significance and 95% confidence intervals (CI). All analyses were carried out in and all figures were produced in Comprehensive Meta-Analysis (CMA) V3.

## RESULTS

The 37 studies included in the analysis had an aggregate of 15,324 participants, 7,661 of which had on-pump CABG and 7,663 had off-pump CABG. Table 1 summarises the characteristics of the included studies. Supplementary Table 1 lists the excluded RCTs and reasons for exclusion.

**Table 1:** Characteristics of the included studies.

Age range (years)	Study	N on CPB (off CPB)	Age on CPB (off CPB)	Male % on CPB (off CPB)	All outcome measures
51-55	Iqbal et al.^[23]^, 2014 Pakistan	100 (100)	53.5 ± 10 (51.6 ± 10.3)	NR	Encephalopathy Hospital stay ICU stay MI Mortality Renal failure Stroke Ventilation time
56-60	Bicer et al.^[24]^, 2014 Turkey	25 (25)	56.9 ± 10.7 (57.7 ± 8.4)	88 (88)	Mortality MDA hs-CRP M30 M65
	Gerola et al.^[25]^, 2004 Brazil	80 (80)	58.9 ± 8.9 (59.1 ± 9.7)	68 (64)	Atrial fibrillation CK-MB Hospital stay ICU stay MI Mortality Stroke
	Kobayashi et al.^[26]^ (JOCRI), 2005 Japan	86 (81)	59 ± 10 (60 ± 7)	86 (87)	Atrial fibrillation CK-MB Graft patency ICU stay MI Mortality Neuron specific enolase S-100 protein Stroke Ventilation time
	Penttila et al.^[27]^, 2001 Finland	11 (11)	59.2 59.5	NR	MI Myocardial markers Myocardial metabolism
61-65	Al-Ruzzeh et al.^[28]^, 2006 UK	84 (84)	63.1 ± 9.6 (63.1 ± 11)	84 (83)	Atrial fibrillation Blood transfusions Graft patency Health-related quality of life Hospital stay ICU stay Mortality Neurocognitive function Stroke Ventilation time
	Angelini et al.^[29]^, 2002 UK	BHACAS 1 100 (100) BHACAS 2 101 (100)	BHACAS 1 61.7 ± 8.6 (62.2 ± 9.6) BHACAS 2 61.2 ± 9.2 (63.8 ± 8.5)	BHACAS 1 79 (82) BHACAS 2 85 (82)	Atrial fibrillation MI Mortality Stroke
	Ascione et al.^[30]^, 2000 UK	100 (100)	63 (63)	79 (82)	Atrial fibrillation Hospital stay ICU stay MI Mortality Stroke Ventilation time
	Fattouch et al.^[31]^, 2009 Italy	65 (63)	61 ± 18 (63 ± 16)	77 (61)	cTnI Cardiac contractile function Hospital stay ICU stay Mortality Ventilation time
	Jongman et al.^[32]^, 2014 The Netherlands	29 (30)	63 (63)	90 (90)	Cardiac failure Inflammatory markers Major bleeding Pulmonary embolism Renal failure Stroke
	Khan et al.^[33]^, 2004 UK	50 (54)	64.7	82 (93)	Blood loss Extubation time Hospital stay ICU stay Infection Low cardiac output MI Mortality Repeat surgery
	Kok et al.^[34]^, 2014 The Netherlands	29 (30)	62.6 ± 9.9 (63 ± 9)	90 (90)	Cerebral oxygenation Cognitive dysfunction Hospital stay ICU stay Stroke
	Légaré et al.^[35]^, 2004 Canada	150 (150)	63.7 ± 10 (62.1 ± 10.1)	79 (81)	Atrial fibrillation Hospital stay ICU stay MI Mortality Stroke Transfusion requirement Ventilation time Wound infection
	Lingaas et al.^[36]^, 2004 Norway	60 (60)	65 ± 8.3 64 ± 7.8	72 (85)	CPB time Ventilation time Reintubation Bleeding Blood transfusions Atrial fibrillation CK-MB Aspartate aminotransferase Stroke Mortality Mediastinitis Graft patency
	Lund et al.^[37]^, 2003 Norway	22 (29)	64 (62)	73.9 (89.7)	Cerebral blood flow Cerebral MRI Neuropsychologic tests Stroke
	Michaux et al.^[38]^, 2011 Switzerland	25 (25)	65 ± 8 (61 ± 9)	84 (84)	Atrial fibrillation cTnI Hospital stay ICU stay MI Mortality Right ventricular function Ventilation time >12 hours
	Motallebzdah et al.^[39]^, 2004 UK	20 (15)	63 (65)	90 (93)	Cerebral blood flow S100 protein Stroke
	Motallebzdah et al.^[40]^, 2007 UK	104 (108)	65.1 ± 0.9 (63.9 ± 0.9)	91 (87)	Cerebral emboli Mortality Neurocognitive function Stroke
	Nathoe et al.^[41]^ (Octopus), 2003 USA	139 (142)	60.8 ± 8.8 61.7 ± 9.2	71 (66)	Cost MI Mortality Quality of life Repeat revascularisation Stroke
	Puskas et al.^[42]^ (SMART), 2003 USA	99 (98)	62.5 ± 9.5 (62.2 ± 11.1)	77 (78)	Atrial fibrillation Coagulopathy and transfusion Hospital stay ICU stay MI Mortality Stroke
	Rastan et al.^[43]^, 2005 Germany	20 (20)	65.3 ± 3.9 (63 ± 6)	80 (80)	CK-MB C-reactive protein cTnI Intraoperative myocardial ischaemia MI Mortality NT-proBNP Oxidative stress Stroke
	Sahlman et al.^[44]^, 2003 Finland	26 (24)	61.5 ± 8.1 (64 ± 9)	77 (88)	Extubation time Bleeding CK-MB ICU stay Hospital stay Weight gain Complement C3 C4 Protein carbonyls Wound infection Low cardiac output syndrome Cerebral infarction Oxidative stress markers
	Shroyer et al.^[45]^ (ROOBY), 2009 USA	1099 (1104)	62.5 ± 8.5 (63 ± 8.5)	99 (99)	Cardiac arrest Coma Hospital stay ICU stay Mediastinitis Mortality New mechanical support Renal failure Reoperation Stroke Tracheostomy Ventilation time
	Straka et al. ^[46]^ (PRAGUE-4), 2004 Czech Republic	184 (204)	62 (63)	86 (77)	Atrial fibrillation Hospital stay ICU stay MI Mortality Renal failure Stroke Ventilation time
	Vedin et al.^[47]^, 2006 ^[47]^ Sweden	37 (33)	65 (65)	84 (78)	Anxiety Cognitive function Depression MI Stroke
66-70	Carrier et al.^[48]^, 2003 Canada	37 (28)	70 ± 6 (70 ± 8)	84 (68)	Mortality MI Stroke Renal insufficiency Respiratory failure/infection Bleeding Blood transfusions ICU stay Hospital stay
	Lamy et al ^[49]^ (CORONARY), 2012 Canada	2377 (2375)	67.5 ± 6.9 (67.6 ± 6.7)	82 (80)	Atrial fibrillation MI Mortality New renal failure Stroke
	Lee et al ^[50]^, 2003Hawaii	30 (30)	66 ± 11.2 (65.5 ± 9.6)	73 (80)	Cerebral microemboli Cerebral perfusion Cost Hospital stay Mortality Neurological function Stroke
	Muneretto et al.^[51]^, 2003 Italy	88 (88)	66 ± 9 (67 ± 8)	59 (63)	Abdominal infarction Atrial fibrillation Hospital stay ICU stay MI Mortality Stroke Ventilation time
	Nesher et al.^[52]^, 2006 Israel	60 (60)	68 ± 5 (67 ± 1)	77 (73)	CK-MB cTnI Cytokines Hospital stay Stroke Ventilation time
	Niranjan et al.^[53]^, 2006 UK	40 (40)			Atrial fibrillation Blood transfusion requirements Clotting tests Hospital stay ICU stay Mortality Postoperative blood loss Stroke Ventilation time
71-75	Hlavicka et al.^[54]^ (PRAGUE-6), 2016 Czech Republic	108 (98)	73.6 ± 7.4 74.7 ± 6.5	57.4 (59.2)	MI Mortality Renal failure Stroke
	Houlind et al.^[55]^ (DOORS), 2012 Denmark	450 (450)	75 (75)	78 (76)	Hospital stay ICU stay MI Mortality Quality of life Stroke
	Lemma et al.^[56]^ (ON-OFF), 2012 Italy	203 (208)	73 (74)	69 (70)	MI Mortality Renal failure Stroke Reoperation for bleeding ARDS
76-80	Diegeler et al. ^[10]^ (GOPCABE), 2013 Germany	1207 (1187)	78.4 ± 2.9 (78.6 ± 3.0)	68 (69)	Hospital stay ICU stay MI Mortality New renal-replacement therapy Repeat revascularisation Stroke Ventilation time
	Møller et al. ^[57]^ (BBS), 2010 Denmark	163 (176)	75.6 ± 4.9 (76.1 ± 5.2)	64 (65)	Cardiac arrest with successful resuscitation Coronary reintervention Low cardiac output syndrome MI Mortality Stroke
	Rogers et al.^[58]^ (CRISP), 2014 UK	53 (53)	75.7 ± 7.7 (76.4 ± 5.8)	76 (78)	MI Mortality Prolonged initial ventilation Renal failure Sternal wound dehiscence Stroke

ARDS=acute respiratory distress syndrome; CK-MB=creatine kinase-muscle/brain; CPB=cardiopulmonary bypass; cTnI=cardiac troponin I; hs-CRP=high-sensitivity creatine phosphate; ICU=intensive care unit; MDA=malondialdehyde; MI=myocardial infarction; MRI=magnetic resonance imaging; NR=not reported; NT-proBNP=N-terminal pro b-type natriuretic peptide; UK=United Kingdom; USA=United States of America

### Stroke Incidence

**Fig. 2:**
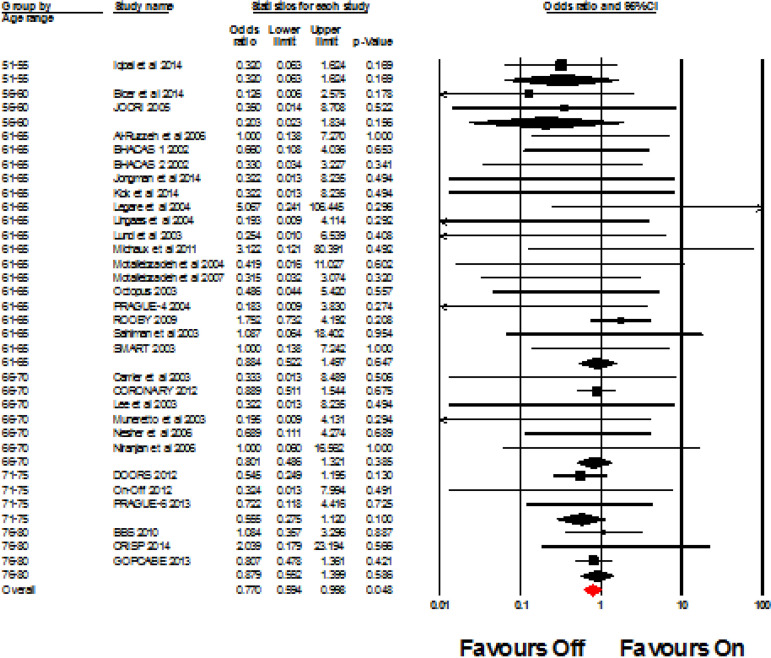
Forest plot for the incidence of stroke. CI=confidence intervals

A total of 31 studies investigated the incidence of stroke. The overall OR was 0.770 (95% CI 0.594, 0.998, I^2^=0%, *P*=0.048). When the patients were grouped according to age, there were no significant differences in the odds of a stroke occurring in the off-pump group compared to the on-pump group. Fifty-one to 55 years old OR 0.32 (95% CI 0.063, 1.624, I^2^=0%, *P*=0.169); 56-60 OR 0.203 (95% CI 0.023, 1.834, I^2^=0%, *P*=0.156); 61-65 OR 0.884 (95% CI 0.522, 1.497, I^2^=0%, *P*=0.647); 66-70 OR 0.801 (95% CI 0.486, 1.321, I^2^=0%, *P*=0.385); 71-75 OR 0.555 (95% CI 0.275, 1.120, I^2^=0%, *P*=0.100); and 76-80 OR 0.879 (95% CI 0.552, 1.399, I^2^=0, *P*=0.586). See Figure 2 for the forest plot.

### Mortality Incidence

**Fig. 3:**
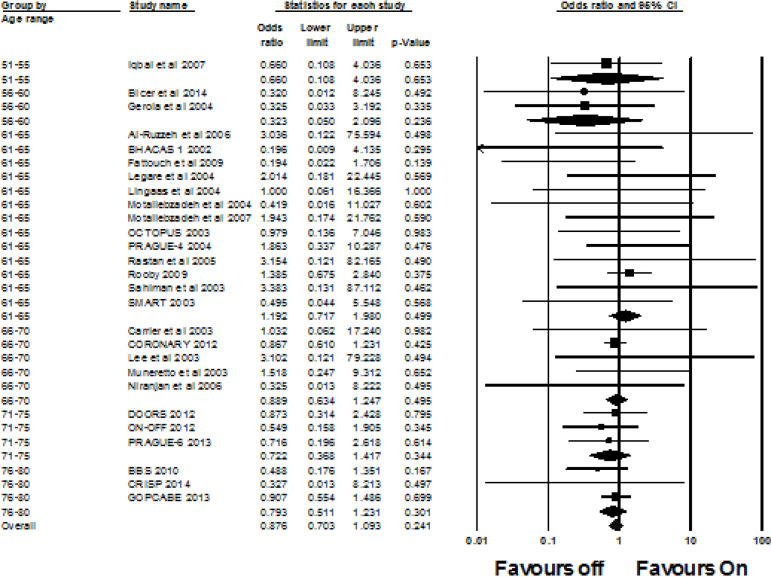
Forest plot for the incidence of mortality. CI=confidence intervals

A total of 27 studies investigated the mortality incidence. The overall OR was 0.876 (95% CI 0.703, 1.093, I^2^=0%, *P*=0.241). There was no significant difference in the odds of mortality occurring in the off-pump group compared to the on-pump group. This was also true when mortality was calculated according to different age groups. Fifty-one to 55 years old OR 0.660 (95% CI 0.108, 4.036, I^2^=0%, *P*=0.653); 56-60 OR 0.323 (95% CI 0.050, 2.096, I^2^=0%, *P*=0.236); 61-65 OR 1.192 (95% CI 0.717, 1.980, I^2^=0%, *P*=0.499); 66-70 OR 0.889 (95% CI 0.634, 1.247, I^2^=0%, *P*=0.495); 71-75 OR 0.722 (95% CI 0.368, 1.417, I^2^=0%, *P*=0.344); and 76-80 OR 0.793 (95% CI 0.511, 1.231, I^2^=0%, *P*=0.301). See Figure 3 for the forest plot.

### Myocardial Infarction Incidence

**Fig. 4:**
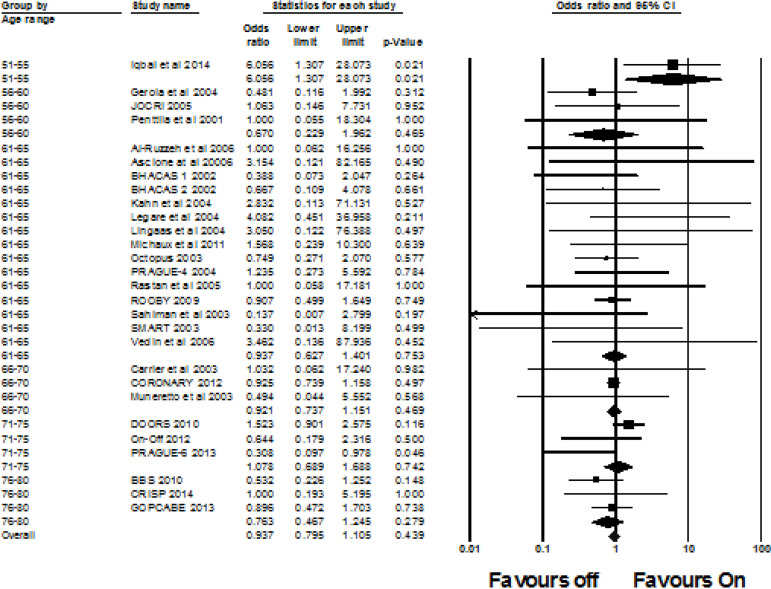
Forest plot for the incidence of myocardial infarction (MI). CI=confidence intervals

A total of 28 studies investigated the MI incidence. The overall OR was 0.937 (95% CI 0.795, 1.105, I^2^=0%, *P*=0.439). There was no difference in the odds of a MI happening in the off-pump group compared to the on-pump group. There was one significant result when patients were grouped according to age band. Fifty-one to 55 years old OR 6.056 (95% CI 1.307, 28.073, I^2^=0%, *P*=0.021); 56-60 OR 0.670 (95% CI 0.229, 1.962, I^2^=0%, *P*=0.465); 61-65 OR 0.937 (95% CI 0.627, 1.401, I^2^=0%, *P*=0.753); 66-70 OR 0.921 (95% CI 0.737, 1.151, I^2^=0%, *P*=0.469); 71-75 OR 1.078 (95% CI 0.689, 1.688, I^2^=70%, *P*=0.742); and 76-80 OR 0.763 (95% CI 0.467, 1.245, I^2^=0%, *P*=0.279). See Figure 4 for the forest plot.

### Risk of Bias

**Supplementary Fig. 2:**
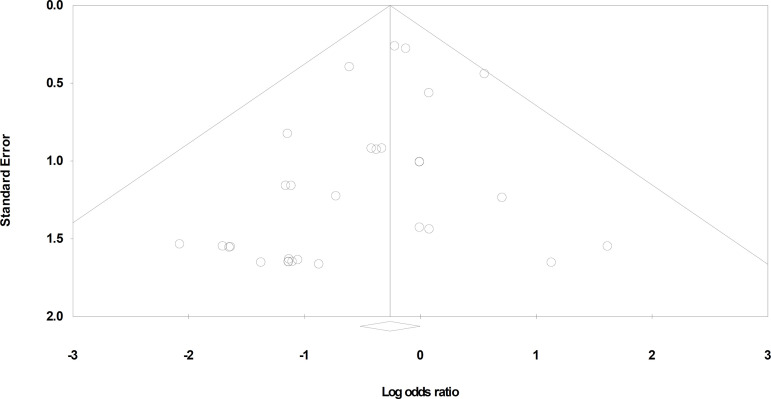
Funnel plot for the incidence of stroke. Begg and Mazumdar’s test (P value): 0.262 and Egger’s test (P value): 0.031.

**Supplementary Fig. 3:**
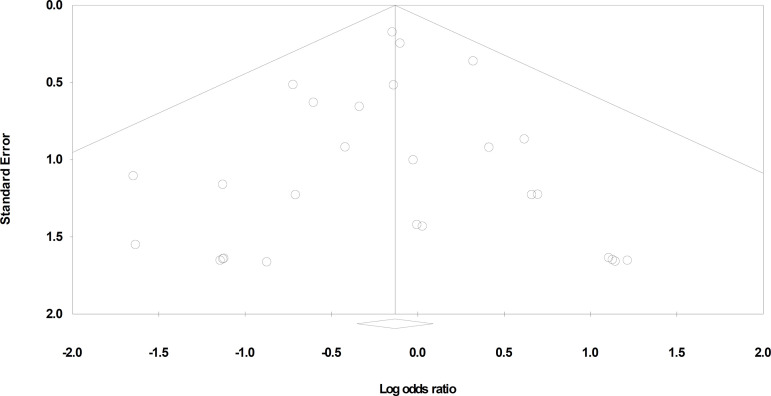
Funnel plot for the incidence of mortality. Begg and Mazumdar’s test (P value): 0.692 and Egger’s test (P value): 0.736.

**Supplementary Fig. 4:**
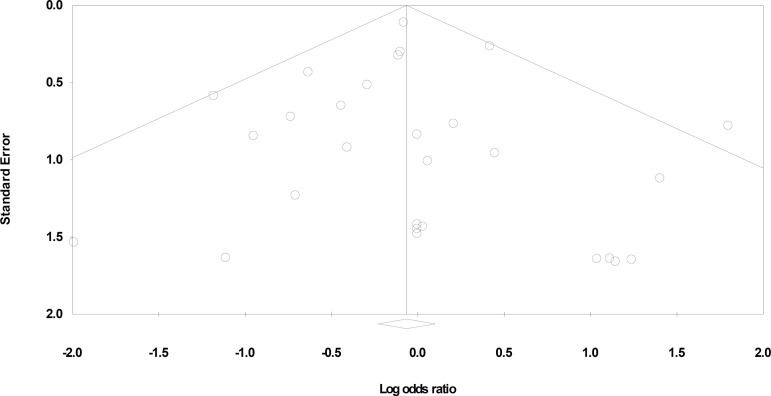
Funnel plot showing the incidence of myocardial infarction. Begg and Mazumdar’s test (P value): 0.167 and Egger’s test (P value): 0.903.

Risk of bias was assessed using a modified Jadad scale with a maximum score of six (Supplementary Table 2). The median score was three. Publication bias was investigated using funnel plots, all of which were symmetrical. The funnel plots with their respective Begg and Mazumdar’s test and Egger’s test statistics can be found in Supplementary Figures 2 to 4.

### Meta-Regression Analyses

**Fig. 5:**
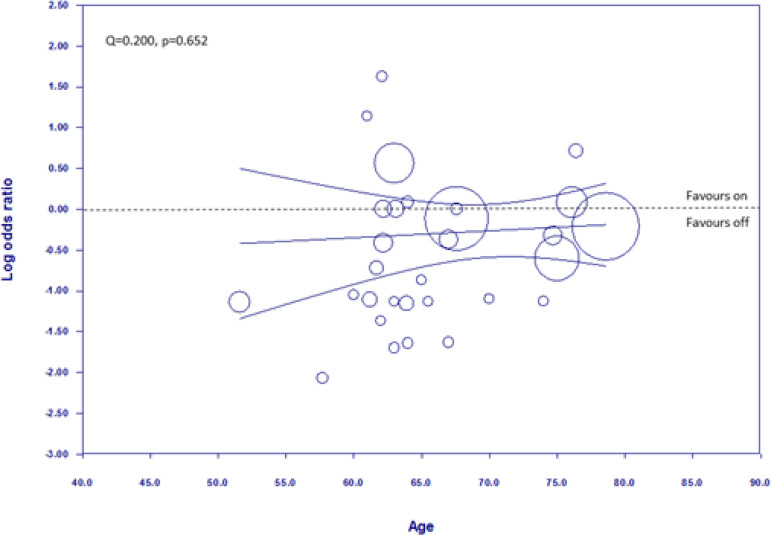
Weighted random-effects meta-regression analysis regressing the log odds ratio (OR) of stroke against age in the off-pump group. Negative values of the log OR mean more benefits for stroke associated with off-pump. The size of the circle corresponds to the inverse variance of the log OR, and thus is related to the statistical weight of the individual trial. The curved lines represent the 95% confidence intervals.

Figure 5 shows the meta-regression plot graphing the log of the OR for stroke occurrence against the mean age of the patients in the off-pump group. The regression line lies slightly on the side favouring off-pump, although the upper 95% CI lies on the side favouring on-pump. There is no difference in the modality favoured across the different ages measured and no relationship between age and the log OR (Q=0.200, *P*=0.652).

**Fig. 6:**
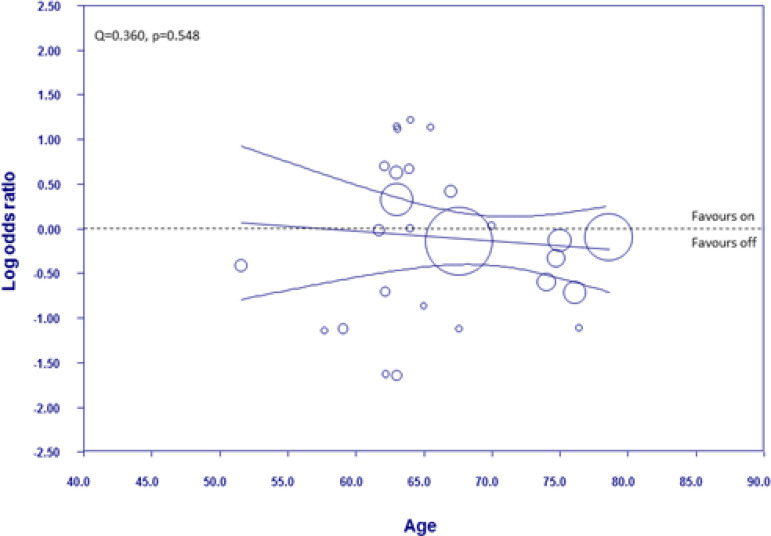
Weighted random-effects meta-regression analysis regressing the log odds ratio of mortality against age in the off-pump group. All other details as in Figure 5.

Figure 6 shows the meta-regression plot graphing the log of the OR for mortality occurrence against the mean age of the patients in the off-pump group. The meta-regression line begins on the side favouring on-pump and then moves to the side favouring off-pump as age increases; however, the 95% CI are equally dispersed either the side of the line of no effect across all the ages. Therefore, there is no difference in the modality favoured across the different ages measured and no relationship between age and the log OR (Q=0.360, *P*=0.548).

**Fig. 7:**
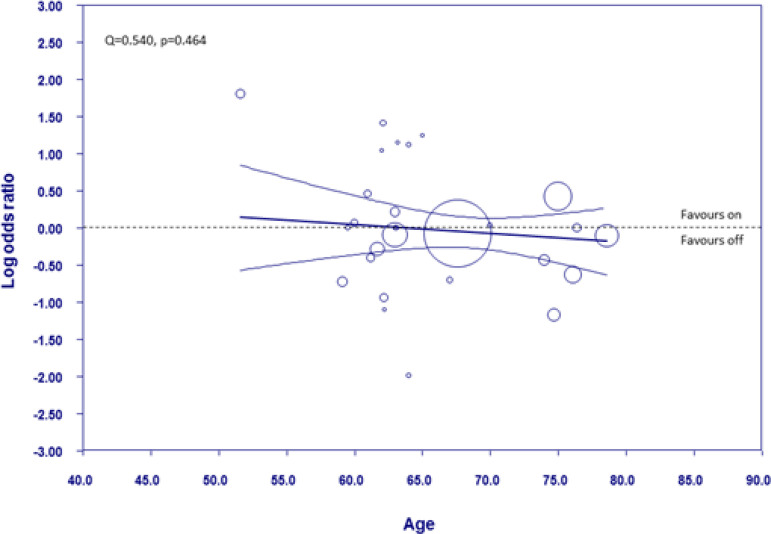
Weighted random-effects meta-regression analysis regressing the log odds ratio of myocardial infarction against age in the off-pump group. All other details as in Figure 5.

Figure 7 shows the meta-regression plot graphing log OR for myocardial infarction occurrence against the mean age of the patients in the off-pump group. The meta-regression line throughout the graph is close to the line of no effect and the 95% CI are equally dispersed about the line of no effect. Therefore, there is no difference in the modality favoured across the different ages measured and no relationship between age and the log OR (Q=0.540, *P*=0.464).

## DISCUSSION

A recent editorial comment^[6]^ suggests that it is important to investigate which category of patient would benefit more from either off- or on-pump CABG. One of the ways in which patients can be categorised is according to age, with patient vulnerability increasing with increasing age. In this novel meta-analysis and meta-regression, we have investigated the effect of age on short-term clinical outcomes following off- or on-pump surgery. Most of the results showed that when patients were classified according to 5-year age bands there was no difference in the OR for stroke, mortality, or MI occurring in the off-pump group compared to the on-pump group. There was a small significant difference in the odds of stroke incidence overall. This was replicated in the meta-regression plots with off-pump favoured for stroke incidence but no differences in the modality favoured according to the different ages measured.

There was no significant difference in the incidence of mortality or MI between on-pump and off-pump CABG overall, mirroring the results of the four largest trials to date^[10,17-19]^ and the three most recent meta-analyses^[2-4]^. This has been the general trend in many studies to date. In addition to this, there was no evidence from this meta-analysis to suggest that the increasing age influences the occurrence of these outcomes as no significant difference in the meta-regression was found. The single exception to this is the MI incidence in the 51-55-year age band; however, it should be noted that this result was based on a single trial and clearly more trials investigating this age group are required.

This meta-analysis found a significantly higher occurrence of stroke in the on-pump group overall but no differences in the different age groupings. The overall result concurs with the results of Deppe et al.^[3]^ and Kowalewski et al.^[2]^. However, they contrast with the four largest trials to date^[10,17-19]^ and the meta-analysis by Dieberg et al.^[4]^. These findings suggest that if there is a difference in the occurrence of stroke between the off-pump and on-pump groups, then age is not the determining factor. In contrast, other retrospective trials, *e.g*. Ricci et al.^[19]^ and the meta-analysis by Altarabsheh et al.^[11]^, examined patients older than 80 years and found lower stroke rates in the off-pump CABG patients. There are no RCTs in patients >80 years old.

It is often hypothesised that off-pump CABG should produce a lower incidence rate of stroke as it does not involve aortic manipulation and cross-clamping^[1]^. But performing the proximal anastomoses during cross-clamping is one possible solution to reduce the aortic manipulation involved in on-pump CABG, thus weakening this hypothesis. There have been many contradictory results as to whether off-pump CABG reduces the risk of stroke and therefore, a definitive answer has not been reached. This could be due to the occurrence of perioperative stroke during CABG being a relatively rare event, meaning that even large trials and meta-analyses lack the weight to support their results. Nevertheless, it is important to continue this evaluation as stroke is a devastating complication of CABG that can lead to a decreased quality of life and increased mortality rate^[20]^. It is important to link potential preoperative risk factors to the incidence of perioperative stroke in order to improve techniques to reduce its occurrence; however, this meta-analysis suggests that age is not one of them. Another potential risk factor that could be associated with an increased risk of stroke is gender. Puskas et al.^[21]^ found that there is a higher incidence of post-operative stroke within the female population, along with a higher mortality and MI rate. They also found that females are more likely to benefit from off-pump CABG than males. Hence, there are many factors that need to be considered and researched further when comparing off-pump and on-pump CABG.

### Study Limitations

Studies scored between two and four out of six on the modified Jadad scale indicating that the median study quality score was moderate (Table 2). There was also some evidence of heterogeneity in many of the studies. Linked to this, not all studies recorded the method of randomisation and there was great variation of methods used between studies. There were also many studies that did not describe dropouts or withdrawals. It is worth noting that it is impossible to use blinding methods within this analysis as surgeons cannot be blinded as to what surgery they are to perform.

One of the most obvious limitations of this study, as in many of the meta-analyses to date that have compared on-pump and off-pump CABG, is the relatively small size of most of the included studies. Only three of the RCTs included more than 1,000 patients^[10,17-18]^ and the next biggest trial included 900 patients^[19-22]^. Many had less than 100 patients (*e.g*., S4) and some as little as <20 patients (*e.g*., S6) within their studies. Removing all studies with <100 patients did not change the overall results, except for the stroke incidence, where the overall significance disappeared. Moreover, the included studies often reported a low occurrence of events in terms of their clinical endpoints, as previously described. This means that most of the included trials were underpowered and endpoints were underestimated, thus the reliability of their results are affected.

In addition, there are many differences in the methods used in each of the included studies. There is variation in the experience of the surgeons and some studies do not state this. For example, one of the larger studies included in this meta-analysis^[17]^ has been criticised for the use of trainee surgeons in their trial who were inexperienced in the off-pump CABG procedure. The CABG procedure itself also varied between studies as some surgeons used hypothermic CPB (*e.g*., S6) whilst others used normothermic CPB (*e.g*., S7). Similarly, there were some variations in the method of cardioplegic arrest used for on-pump CABG; some trials used cold blood cardioplegia (*e.g*., S13) and some used warm blood cardioplegia (*e.g*., S9).

Another big limitation of this study is the small number of trials with a mean age between 51-55 years or >66 years, meaning that these age groups were underpowered compared to the others. On top of this, there were no trials with a mean age of over 80 years, meaning that this age group was completely omitted from the analysis. In order to gain a better analysis of the effect that age has on the outcomes of on-pump and off-pump CABG, more trials need to be completed, including patients within these age groups.

## CONCLUSION

There is continuing debate as to which approach on- or off-pump CABG is superior. There are many ways in which patients could be subdivided to discover which selected groups would benefit most from one approach or another, including age. This meta-analysis and meta-regression has shown that separating patients according to their age up to the age of 80 years does not affect whether off-pump or on-pump should be favoured in these patients.

**Table:** 

Authors' roles & responsibilities	
HM	Substantial contributions to the conception or design of the work; or the acquisition, analysis, or interpretation of data for the work; final approval of the version to be published.
GD	Substantial contributions to the conception or design of the work; or the acquisition, analysis, or interpretation of data for the work; final approval of the version to be published.
NS	Substantial contributions to the conception or design of the work; or the acquisition, analysis, or interpretation of data for the work; final approval of the version to be published.
NK	Substantial contributions to the conception or design of the work; or the acquisition, analysis, or interpretation of data for the work; final approval of the version to be published.

## Figures and Tables

**Figure:**
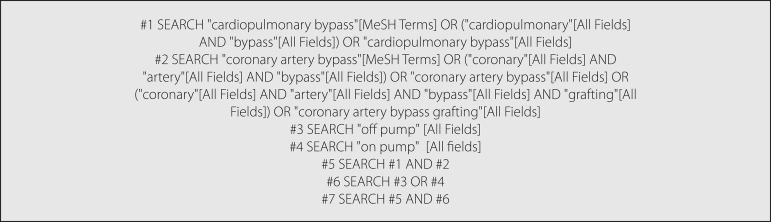
Pubmed Search Strategy.

## Supplementary Fig. 1

**Supplementary Table 1:** Excluded studies and reasons for exclusion.

Study	Reason
Chowdhury et al., 2008	Mean age crossed over two age groups
Covino et al., 2001	Did not record mean age
Formica et al., 2013	Mean age crossed over two age groups
Gulielmos et al., 2000	Mean age crossed over two age groups
Hernandez et al., 2007	Did not record mean age
Hoel et al., 2007	Did not record mean age
Kobayashi et al., 2005	Mean age crossed over two age groups
Kochamba et al., 2000	Mean age crossed over two age groups
Kunes et al., 2007	Mean age crossed over two age groups
Medved et al., 2008	Mean age crossed over two age groups
Paparella et al., 2006	Did not record mean age
Rachwalik et al., 2006	Mean age crossed over two age groups
Rainio et al., 2007	Mean age crossed over two age groups
Raja et al., 2003	Did not record mean age

**Supplementary Table 2:** Examination of study quality.

Study	Randomisation	Methods of randomisation	Methods of blinding described	Method of blinding appropriate	Withdrawals/dropouts described	Other potential bias	Score (out of 6)
Al-Ruzzeh et al.^[28]^, 2006	Yes	Yes	No	N/A	No	No	3
Angelini et al.^[29]^, 2002	Yes	Yes	No	N/A	No	Yes	2
Ascione et al.^[30]^, 2000	Yes	Yes	No	N/A	No	No	3
Bicer et al.^[24]^, 2014	Yes	No	No	N/A	No	No	2
Carrier et al.^[48]^, 2003	Yes	No	No	N/A	No	No	2
Diegeler et al.^[10]^, 2013	Yes	Yes	No	N/A	Yes	No	4
Fattouch et al.^[31]^, 2009	Yes	Yes	No	N/A	No	No	3
Gerola et al.^[25]^, 2004	Yes	No	No	N/A	No	No	2
Hlavicka et al.^[54]^, 2013	Yes	Yes	No	N/A	Yes	No	4
Houlind et al. ^[22]^, 2012	Yes	Yes	No	N/A	Yes	No	4
Iqbal et al.^[23]^, 2014	Yes	No	No	N/A	No	No	2
Jongman et al.^[32]^, 2014	Yes	No	No	N/A	No	No	2
Khan et al.^[33]^, 2004	Yes	No	No	N/A	No	No	2
Kobayashi et al.^[26]^, 2005	Yes	Yes	No	N/A	No	No	3
Kok et al.^[34]^, 2014	Yes	No	No	N/A	Yes	No	3
Lamy et al.^[49]^, 2012	Yes	Yes	No	N/A	No	No	3
Lee et al.^[50]^, 2003	Yes	Yes	No	N/A	No	No	3
Légaré et al.^[35]^, 2004	Yes	Yes	No	N/A	No	Yes	2
Lemma et al.^[56]^, 2012	Yes	Yes	No	N/A	Yes	No	4
Lingaas et al.^[36]^, 2004	Yes	No	No	N/A	No	No	2
Lund et al.^[37]^, 2003	Yes	No	No	N/A	Yes	No	3
Michaux et al.^[38]^, 2011	Yes	Yes	No	N/A	No	Yes	2
Moller et al.^[57]^, 2010	Yes	Yes	No	N/A	No	Yes	2
Motallebzadeh et al.^[39]^, 2004	Yes	Yes	No	N/A	No	No	3
Motallebzadeh et al.^[40]^, 2007	Yes	Yes	No	N/A	No	No	3
Munereto et al.^[51]^, 2003	Yes	No	No	N/A	No	No	2
Nathoe et al.^[41]^, 2003	Yes	Yes	No	N/A	No	No	3
Nesher et al.^[52]^, 2006	Yes	Yes	No	N/A	Yes	No	4
Niranjan et al.^[53]^, 2006	Yes	Yes	No	N/A	No	No	3
Penttila et al.^[27]^, 2001	Yes	No	No	N/A	No	No	2
Puskas et al.^[42]^, 2003	Yes	Yes	No	N/A	Yes	Yes	3
Rastan et al.^[43]^, 2005	Yes	Yes	No	N/A	No	No	3
Rogers et al.^[58]^, 2014	Yes	Yes	No	N/A	Yes	Yes	3
Sahlman et al.^[44]^, 2003	Yes	No	No	N/A	No	No	2
Shroyer et al.^[45]^, 2009	Yes	Yes	No	N/A	Yes	Yes	3
Straka et al.^[46]^, 2004	Yes	Yes	No	N/A	Yes	No	4
Vedin et al.^[47]^, 2006	Yes	No	No	N/A	Yes	No	3

Median score=3. N/A=not available
